# Biophysical chemistry behind sickle cell anemia and the mechanism of voxelotor action

**DOI:** 10.1038/s41598-024-52476-8

**Published:** 2024-01-22

**Authors:** Mohd. Suhail

**Affiliations:** Department of Chemistry, Siddhartha (PG) College, Aakhlor Kheri, Deoband (Saharanpur), Uttar Pradesh 247554 India

**Keywords:** Biochemistry, Biophysics, Biotechnology, Cell biology, Chemical biology, Computational biology and bioinformatics, Molecular biology, Molecular medicine, Chemistry

## Abstract

Sickle cell anemia disease has been a great challenge to the world in the present situation. It occurs only due to the polymerization of sickle hemoglobin (HbS) having Pro–Val–Glu typed mutation, while the polymerization does not occur in normal hemoglobin (HbA) having Pro–Glu–Glu peptides. It is also well confirmed that the oxygenated HbS (OHbS) does not participate in the polymerization, while the deoxygenated HbS (dHbS) does, which causes the shape of red blood cells sickled. After polymerization, the blood has a low oxygen affinity. Keeping this fact into consideration, only those drugs are being synthesized that stabilize the OHbS structure so that the polymerization of HbS can be stopped. The literature data showed no systematic description of the changes occurring during the OHbS conversion to dHbS before polymerization. Hence, an innovative reasonable study between HbA and HbS, when they convert into their deoxygenated forms, was done computationally. In this evaluation, physiochemical parameters in HbA/HbS before and after deoxygenation were studied and compared deeply. The computationally collected data was used to understand the abnormal behaviour of dHbS arising due to the replacement of Glu6 with Val6. Consequently, during the presented computational study, the changes occurring in HbS were found opposite/abnormal as compared to HbA after the deoxygenation of both. The mechanism of Voxelotor (GBT-440) action to stop the HbS polymerization was also explained with the help of computationally collected data. Besides, a comparative study between GBT-440 and another suggested drug was also done to know their antisickling strength. Additionally, the effect of pH, CO, CO_2_, and 2,3-diphosphoglycerate (2,3-DPG) on HbS structure was also studied computationally.

## Introduction

Sickle cell anemia disease (SCAD) is a molecular disease^1^ that occurs due to the gene mutation^2^. The low oxygen affinity of a mutant hemoglobin is a characteristic of SCAD^3,4^. It has already been confirmed^2^ that one of the glutamic acid residues of Pro–Glu–Glu (PGG) present in normal hemoglobin (HbA) is replaced by valine residue i.e. Pro–Val–Glu (PVG) in sickle cells, also called E6V mutation. Although sickle hemoglobin (HbS) has normal oxygen binding affinity, its polymerized form does not^5^. The production of abnormal Red Blood Cells (RBCs) under hypoxic conditions^4–8^ and low HbS solubility^9,10^ are just because of the polymerization of the same^5–10^. Actually, the deoxygenated form of HbS (dHbS) takes part in the polymerization reaction rapidly while its oxygenated form (OHbS) does not^7^. Keeping this fact into consideration, the OHbS is made pharmacologically more stable so that the HbS polymerization can be inhibited to prevent RBC sickling and provide long-term disease modification^11–13^. It must be remembered that the polymerization of a biomolecule involves two steps. The first is the conformational change of the native monomer; the second is the binding of the ‘open’, flipped, monomer to the growing interdigitating polymer^14^. Although different researchers have given different perspectives associated with SCAD and the polymerization of sickle hemoglobin, they did not give a deep insight which is why their studies raise many questions. Different aspects in HbA/HbS evaluation with their gaps are as follows:

### Biophysical interaction-based evaluation

The steric and electrostatic effects of the residue hosted through the mutation^15^ have also been found as^7,15–17^ a reason for the low oxygen affinity of a mutant hemoglobin. As per the fundamental electrostatic analysis^16,17^, it has been confirmed that the effect/involvement of E6V mutation in the polymerization of dHbS is not direct. This is because Glu6 itself does not seem to be engaged in any significant electrostatic interaction with Hb’s interacting partners^18^. Hence, the loss of Glu6’s side chain leads to indirect local electrostatic equilibrium perturbation as per intermolecular electrostatic interactions. These intermolecular interactions include salt bridges, hydrogen bonds, and charge-dipole interaction which may stabilize^19–21^ or destabilize^22,23^ biomolecules^24–26^. This electrostatic equilibrium perturbation is of functional significance for the overall structural stability of Hb^17^. Moreover, the presence and strength of the salt bridge make the Hb molecule stable which was proved when a different form of HbS^27^ was observed after the substitution of Lys132 with Asn132. It is because Lys132 forms a Lys132-Glu7 typed salt bridge with Glu7 but no salt bridge is formed between Asn132 and Glu7 because Asn132 carries no charge. Therefore, the interruption in the salt bridge caused by the Asn-Lys substitution directly affected the α_2_β_2_ stability, underlining the disquiet of local electrostatic equilibrium by SCD-linked mutations such as Lys132Asn and E6V too. The same thing was also supported by the earlier study of molecular dynamics simulation confirming the inter-facial electrostatic collaborations accountable for HbS polymerization^7,28^. Literature data shows the lack of comparative study between OHbS and dHbS including the variation occurring in biophysical interactions along with the OHbS conversion into dHbS before polymerization. Besides, the effect of the existence of not only 2,3-diphosphoglycerate (2,3-DPG) but also the anti-sickling drugs (GBT440) on biophysical interactions in HbS was found out of expectations. Keeping these facts into consideration, the biophysical interaction-based mechanism of GBT440 action was explained in the presented article. Besides, the same article also gives the biophysical interaction-based explanation of how this sickle anemia-linked E6V mutation causes the sickling of RBC via dHbS polymeirzation.

### Nature-based evaluation

Quantum level study has clearly shown that the PVG has a more reactive nature to take part in biochemical reactions as compared to PGG^29^. Moreover, the normal Pro–Glu–Glu peptides are hydrophilic in nature, while the mutant Pro–Val–Glu peptides are hydrophobic due to the presence of hydrophobic valine residue^30–32^. Hence, researchers^30–32^ have also suggested that the hydrophobic R-group of Val-beta6 (E6V) appears to insert into a hydrophobic pocket constituted by β88 leucine (Leu-beta88), β85phenylalanine (Phe-beta85), and β73 aspartic acid (Asp-beta73) residues on adjacent deoxygenated sickle hemoglobin molecules (dHbS-M)^33,34^. For the insertion of the R-group of Val-beta6 in the acceptor pocket regions on adjacent dHbS-M, not only stereospecificity to the Leu-beta88 side chain^30^ but also the contact position on dHbS-M was found favourable^30–34^. On the other hand, no such favourable conditions were noted for the insertion of the hydrophilic R-group of Glu-beta6 (β-glutamic acid) into the same which is why polymerization does not occur in deoxyHbA^35^. This thing does not give any correlation with electrostatic forces present in HbS/HbA. It is because the nonpolar suppressed surface area reflects many things such as the hydrophobic effect, the major driving potency in protein binding and folding, but to flexible extents^36,37^. Besides, PVG and PGG both are present on the surface, and both can make salt bridges for hemoglobin stability because the salt bridges present on the surface provide stability to protein^38^. The presented article explains why a hydrophobic residues i.e. PVG inserts into the neighboring hydrophobic pockets despite providing stability to a protein via salt bridge formation.

### Medium-based evaluation

Like the studies described above, the effect of an acidic/basic medium on dHbS polymerization has great importance whose appropriate and deep explanation still needs confirmation. Different studies were done to understand the effect of pH, carbon monoxide, and other factors on HbS polymerization. For example, the main findings regarding the pH effect showed that the lowered pH over the physiological range promotes not only HbS polymerization but also RBC sickling^39^. Hence, the chance for polymerization of HbS is increased at lower pH. The exact mechanism by which protons (H^**+**^) facilitate the HbS polymerization to cause a lowering in oxygen affinity at low pH is not known^39^. Moreover, the decrease in hemoglobin affinity for oxygen caused by the accumulation of 2,3-DPG (2,3-diphosphoglycerate) has also been found to correlate with pH^10,40^. This mechanism for the intracellular accumulation of 2,3-DPG (2,3-diphosphoglycerate) increases oxygen release from RBCs under circumstances where it is needed most^41^. This release is potentiated by the Bohr Effect, in which hemoglobin‘s binding affinity for oxygen is also reduced because of the high concentration of carbon dioxide. In tissues with high energetic demands, oxygen is rapidly consumed, which increases the concentration of H^**+**^ and carbon dioxide^42^. It is well confirmed that the probability of RBCs sickling is also increased due to the higher intracellular concentration of 2,3-DPG in RBC hemoglobin^43,44^. Of course, the deoxygenated form of HbS takes part in polymerization reaction rapidly, but it must be remembred that the oxygen affinity and hemoglobin polymerization both are different things. It is because the loss of oxygen or less oxygen affinity is related to the entry of 2,3-DPG in hemoglobin structure^45–47^, while the polymerization reaction is related to the RBC sickling caused by residual combination in hemoglobin^48,49^. The most important thing noted in the literature data was the lower oxygen affinity of hemoglobin caused by the accumulation of 2,3-DPG at higher pH^10^, while at lower pH, the RBC sickling was found promoted^39^. The 2,3-DPG entry in RBCs can occur easily if the intracellular medium is slightly basic^10,50^ which is why its entry is found pH dependent. The presented computationally-evaluated literature data explains how a decrease in intracellular pH is responsible for (i) the removal of 2,3-DPG from CO_2_-added medium, and (ii) the promotion of HbS polymerization. Besides these facts, (i) the HbS polymerization caused by the 2,3-DPG accumulation at higher pH, and (ii) the reason for hemoglobin stability caused by the addition of carbon monoxide gas are also explained deeply.

### Anti-sickling drugs and their function

**Figure 1:**
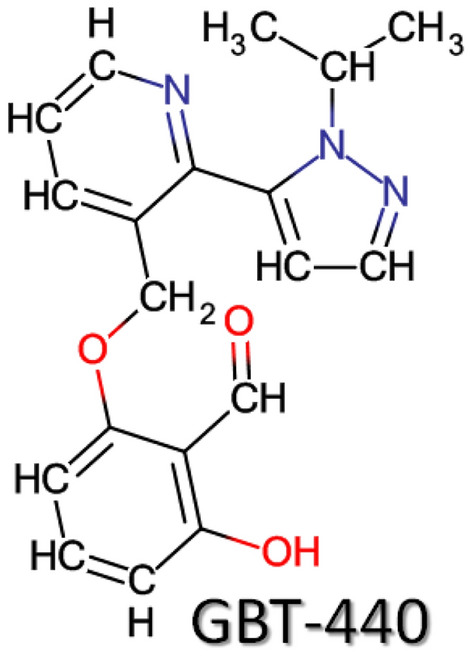
The structure of voxelotor, an anti-sickling drug.

Voxelotor (GBT440), the first hemoglobin oxygen-affinity modulator^51^, when bound to HbS, inhibits polymerization and red blood cell sickling^52^. Therefore, the U.S. FDA has approved GBT440 (Fig. 1) as an anti-sickling agent^53^. The literature data related to the changes in HbS biophysical interactions caused by GBT440 (voxelotor) was not found yet. Hence, a satisfactory explanation of the mechanism of GBT440 action is given here after reading its mechanism-related articles^12,53–66^. Besides, the presented article also describes the biophysical changes occuring in voxelotor-treated HbS structure.

Besides GBT440, many drugs^11,12,53–57^ including VZHE-039 have also been synthesized. The most common point in the mechanism of antisickling drugs^11,12,53–57^ is the formation of Schiff base adduct. For this, a condensation reaction occurs between aldehyde moiety of anti-sickling drug^11,12,53–57^ and αVal1 N-terminal amines. It was very much interesting to note that the mutation point is present in the beta chain of HbS, while the available/synthesized anti-sickling drugs attach to the alpha chain of HbS. Moreover, a systematic mechanism of voxelotor to make the OHbS stable is not described till now, but for the first time, a computationally evaluated and acceptable mechanism for the same is described here. Besides, VZHE-039 data (PDB ID: 6xd9^53^) was also used just for the determination of its strength to make OHbS stable in comparison to voxelotor. Hence, the data regarding VZHE-039 was not evaluated as considerably as GBT-440 in the presented computational study.

## Results

The results of the computationally evaluated literature data describe all aspects mentioned in the introductory part. The SCAD-associated interesting facts evaluated computationally are given below.

### Physiochemical parameters

Although there were many physiochemical interactions among polypeptide chains of HbA and HbS, only those physiochemical interactions were studied deeply which were linked to PGG and PVG residues. The stability at the mutation point of B, D, F, and H chains of dHbS was evaluated by comparing it with the same chains of OHbA/dHbA. It was observed that the PVG residues present in ‘F and H’ β-chains of dHbS were found as stable as those of OHbA. This is because the distance of electrostatic interactions made by PVG residues in ‘F and H’ β-chains of dHbS was found similar to the PGG residues of the same OHbA β-chains (Table 1 and Table 2). Moreover, the salt bridge number and their strength determine the stability of Hb protein as per section “Biophysical interaction-based evaluation” of the presented article. Hence, after deoxygenation, the PVG residues in ‘F and H’ β-chains of HbS attain the same stability as PGG do in OHbA i.e. no major changes occur in both chains of HbS after deoxygenation.

**Table 1:** Physiochemical interactions involved in PGG of HbA.

PDB ID (HbA type)	Interactions		Residues		Distance in A°
	Category	Type	From	To	
1hho (OHbA)	Conventional H-Bond	hydrogen bond	B:SER9:N	B:PRO5:O	2.70
			B:SER9:N	B:GLU6:O	3.23
			B:ALA10:N	B:GLU6:O	3.15
			B:THR4:N	B:GLU7:OE2	3.19
			B:GLU7:N	B:THR4:O	3.05
	Salt bridge	hydrogen bond; electrostatic	B:LYS132:NZ	B:GLU7:OE2	3.57
			D:LYS132:NZ	None	Nil
			F:LYS132:NZ	F:GLU7:OE1	2.77
			F:LYS132:NZ	F:GLU7:OE2	2.77
			H:LYS132:NZ	H:GLU7:OE1	2.96
2m6z (OHbA*)	Salt bridge	hydrogen bond; electrostatic	B:LYS132:HZ3	B:GLU7:OE1	2.37
			B:LYS132:HZ2	B:GLU7:OE1	2.37
			D:LYS132:HZ3	D:GLU7:OE1	2.37
			D:LYS132:HZ2	D:GLU7:OE1	2.37
1a3n (dHbA)	Carbon H-Bond	hydrogen bond	B:PRO5:CD	B:GLU6:OE1	3.33
	Conventional H-Bond	hydrogen bond	B:SER9:OG	B:PRO5:O	3.12
			B:SER9:N	B:PRO5:O	2.99
			B:ALA10:N	B:GLU6:O	2.90
			B:SER9:N	B:GLU6:O	3.37
			B:GLU6:N	B:THR4:OG1	3.32
			B:ALA10:N	B:GLU7:O	3.20
			B:VAL11:N	B:GLU7:O	2.91
			B:PRO5:CD	B:GLU6:OE1	3.33
			B:THR4:N	B:GLU7:OE1	2.85
			B:GLU7:N	B:THR4:OG1	2.99
	Salt bridge	hydrogen bond; electrostatic	B:LYS132:NZ	B:GLU7:OE1	2.91
			D:LYS132:NZ	D:GLU7:OE1	2.89

*Oxygenated with CO.

**Table 2:** Physicochemical interactions involved in PVG of HbS.

PDB ID (HbS type)	Interactions		Residues		Distance in A°
	category	Type	from	To	
5e6e (OHbS*)	Water H-Bond; Conventional H-Bond	hydrogen bond	B:PRO5:CD	B:HOH473:O	3.78
			B:HOH319:O	B:VAL6:O	2.63
	Conventional H-Bond	hydrogen bond	B:SER9:N	B:PRO5:O	2.77
			B:LYS8:N	B:PRO5:O	3.29
			B:VAL6:N	B:THR4:OG1	3.36
			B:SER9:N	B:VAL6:O	3.26
			B:ALA10:N	B:VAL6:O	2.87
			B:GLU7:N	B:THR4:OG1	3.06
			B:GLU7:N	B:THR4:O	2.96
			B:VAL11:N	B:GLU7:O	2.91
			B:ALA10:N	B:GLU7:O	3.14
	Salt bridge	hydrogen bond; electrostatic	B:LYS132:NZ	B:GLU7:OE1	2.63
5e83 (Treated OHbS*)	Salt bridge	hydrogen bond; electrostatic	B:LYS132:NZ	B:GLU7:OE2	3.26
			B:LYS132:NZ	B:GLU7:OE1	3.27
			D:LYS132:NZ	D:GLU7:OE2	2.78
	Salt bridge**	hydrogen bond; electrostatic	B:LYS82:NZ	D:HIS146:O	3.24
6xd9 (Treated OHbS*)	Salt bridge	hydrogen bond; electrostatic	B:LYS132:NZ	B:GLU7:OE2	3.55
			B:LYS132:NZ	B:GLU7:OE1	3.27
			D:LYS132:NZ	D:GLU7:OE2	2.80
2hbs (dHbS)	Carbon H-Bond	hydrogen bond	B:SER9:OG	B:PRO5:O	2.93
			B:SER9:N	B:PRO5:O	2.83
			B:LYS8:N	B:PRO5:O	3.24
			B:THR4:OG1	B:GLU7:OE1	3.39
			B:THR4:N	B:GLU7:OE1	2.96
			B:ALA10:N	B:VAL6:O	2.99
			B:VAL11:N	B:GLU7:O	2.88
			B:GLU7:N	B:THR4:O	2.63
	Water H-Bond; Conventional H-Bond	hydrogen bond	B:HOH276:O	B:GLU7:OE2	3.13
	Salt bridge	hydrogen bond; electrostatic	B:LYS132:NZ	B:GLU7:OE2	3.18
			B:LYS132:NZ	B:GLU7:OE1	3.22
			D:LYS132:NZ	None	Nil
			F:LYS132:NZ	F:GLU7:OE1	2.77
			F:LYS132:NZ	F:GLU7:OE2	2.90
			H:LYS132:NZ	H:GLU7:OE2	2.96

*Oxygenated with CO; **Additional salt bridge.

On the opposite side, the PVG residues were found not to provide the stability to ‘B and D’ β-chains of dHbS as PGG does to OHbA/dHbA. This is because the distance of the electrostatic interactions made by PVG residues in ‘B and D’ β-chains of dHbS was found very different from the PGG residues of the same dHbA β-chains (Table 1 and Table 2). Moreover, other structural differences between HbA and HbS were also found at the dissimilar points (PGG and PVG) of the ‘B and D’ β-chains of HbA and HbS. Therefore, keeping these facts into consideration, the biophysical interactions made by PGG and PVG were compared only in ‘B and D’ β-chains of HbA and HbS respectively. The number of salt bridges made by PGG residues of β-chain (B chain) was found 1 in both OHbA and dHbA with a distance of 3.57 A° and 2.91 A° respectively (Table 1). On the other hand, the number of salt bridges made by PVG residues of β-chain (B chain) was 1 in OHbS and 2 in dHbS with the distance of 2.63 A° and 3.18 A° & 3.22 A° respectively (Table 2). Furthermore, 1 salt bridge made by PGG residues of β-chain (D chain) was found in dHbA with a distance of 2.89 A° (Table 2), while no salt bridge was found at the mutation point (PGG) of the same dHbS chain (Table 2).

Besides the salt bridge, other types of interactions involved in Pro–Glu–Glu and Pro–Val–Glu residues in ‘B and D’ β-chains of HbA and HbS are also given in Table 1 and Table 2 respectively. It was found that the number of hydrogen bonds and their distance changed when OHbA/OHbS structures were compared with dHbA/dHbS (Table 1 and Table 2). On the other hand, the distinguished and extra salt bridge (B:LYS82:NZ-D:HIS146:O) was found (Table 2) in the β-chain of OHbS structure containing the anti-sickling drug, GBT440 (PDB ID: 5e83^13^). In this additional salt bridge, the ‘Lys residue’ present in ASN-LEU-LYS-GLY-THR peptides of OHbS β-chain i.e. ‘B’ chain (5e83^13^) was found attached to the ‘HIS residue’ of the adjacent β-chain (D chain). Such type of salt bridge was not found in the same β-chains of OHbA, dHbA, OHbS, and dHbS (pdb codes: 1hho^67^, 1a3n^68^, 5e6e^69^ and 2hbs^70^ respectively). Moreover, this type of additional salt bridge was also not found in VZHE-039 containing HbS protein (6xd9^53^). In the case of VZHE-039 treated HbS, the distance of one of the two salt bridges in PVG residues was found the same but that of another one was found longer (Table 2) when compared with GBT-440 treated HbS (5e83^13^). Moreover, the number of salt bridges on β-chain (B chain) was found greater in the hemoglobin (Hb) oxygenated with CO (2m6z^71^), as compared to the hemoglobin oxygenated with oxygen (1hho^67^) (Table 1).

### Peptide bond torsion

**Figure 2:**
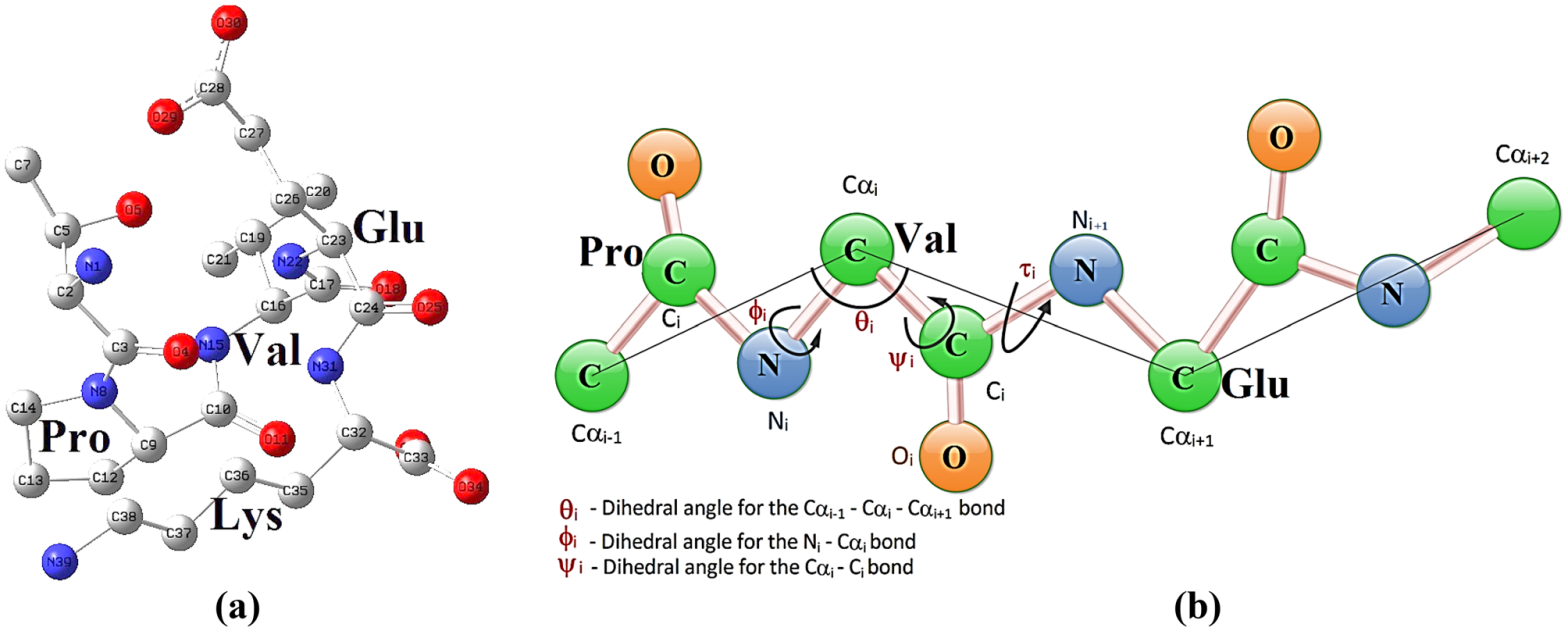
(**a**) Atoms tagged for torsion angle study in HbS, and (**b**) torsion angles designed for Pro–Val and Val–Glu peptide bond in (**a**).

**Figure 3:**
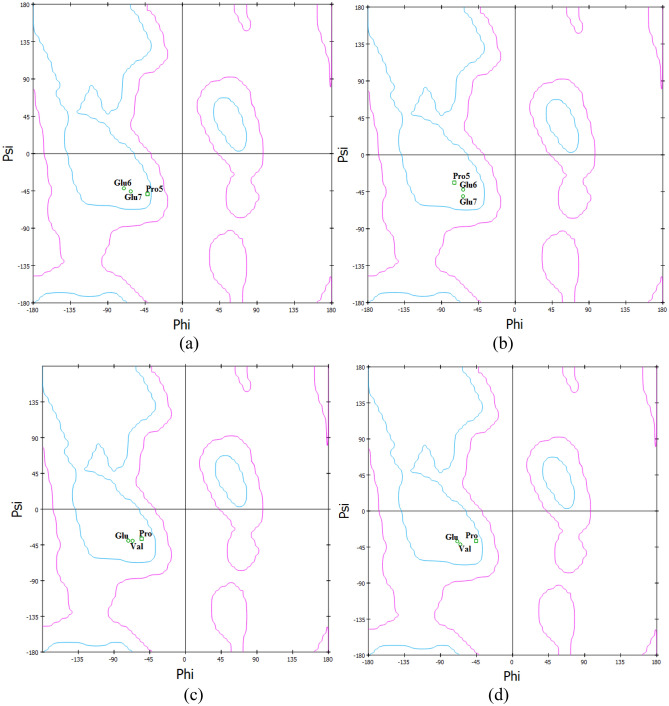
Ф (phi) and Ѱ (psi) bonds torsion found in PGG of (**a**) OHbA, (**b**) dHbA, and PVG of (**c**) OHbS, and (**d**) dHbSin β-chain (B chain)

The variation in peptide bond torsion also gave very significant points. A smooth variation in the peptide bond torsion was noted when all atoms present in PVG were tagged (Fig. 2) for the same (Tables 3, 4). It was noticed that the peptide bond torsion in Pro–Glu and Glu–Glu increases when oxygenated HbA converts into its deoxygenated form (Table 4). On the other hand, the peptide bond torsion in Pro–Val increases but that in Val–Glu decreases when oxygenated HbS (OHbS) converts into its deoxygenated form (dHbS). All these things were also noted (Table 4) in the case of TOHbS (oxygenated HbS treated with GBT440) so that the mechanism of action of the anti-sickling drug could be explained. Moreover, in the main findings observed during the structural evaluation of OHbA, dHbA, OHbS, and dHbS, the Ramachandran plot-based study of both residues (Pro–Glu–Glu and Pro–Val–Glu) was also found very supportive (Fig. 3a–d). Based on the Ramachandran plot study, when OHbA converts into dHbA, the value of Ф (phi) bond is decreased for Pro5, and that of Ѱ (psi) for the same increased, while only minor changes occur in values of Ф (phi) and Ѱ (psi) bonds for Glu6 and Glu7 (Fig. 3a,b). Based on the same study, when HbS converts into dHbS, the value of Ѱ (psi) bond is increased for Glu7, and that of Ф (phi) for the same includes very little change, while no major changes occur in values of Ф (phi) and Ѱ (psi) bonds for Pro5 and Val6 (Fig. 3c,d).

**Table 3:** Dihedral angles involved in Pro–Val–Glu peptide bonds (Fig. 2b) with atoms tagged in HbS (Fig. 2a).

HbS	Tag	Symbol	Connectivity with tagged atom			Bond	Angle	Dihedral	Coordinates		
									X	Y	Z
OHbS (5e6e)	8	N	3	2	1	1.3	117.49	163.97	15.36	40.99	69.05
	9	C*	8	3	2	1.46	120.38	179.33	15.97	42.06	69.84
	10	C	9	8	3	1.53	111.13	− 55.22	17.24	41.57	70.56
	11	O	10	9	8	1.22	120.64	141.92	18.21	42.30	70.67
	12	C	9	8	3	1.52	102.60	− 175.25	14.85	42.41	70.83
	13	C	12	9	8	1.49	102.14	− 38.34	13.63	42.30	69.97
	14	C	8	3	2	1.47	126.14	− 19.53	13.89	41.03	69.13
	15	N	10	9	8	1.33	116.29	− 37.28	17.21	40.32	71.02
	16	C*	15	10	9	1.45	121.43	− 178.88	18.34	39.75	71.73
	17	C	16	15	10	1.51	109.12	− 66.57	19.51	39.61	70.78
	18	O	17	16	15	1.23	120.05	139.84	20.65	39.88	71.17
	19	C	16	15	10	1.54	112.78	172.29	18.03	38.36	72.34
	20	C	19	16	15	1.51	110.09	167.52	19.31	37.69	72.80
	21	C	19	16	15	1.54	109.55	− 72.75	17.09	38.53	73.56
	22	N	17	16	15	1.33	116.96	− 39.93	19.24	39.21	69.54
	23	C*	22	17	16	1.45	119.78	179.82	20.32	39.06	68.58
	24	C	23	22	17	1.53	109.15	− 72.03	20.82	40.45	68.16
	25	O	24	23	22	1.23	120.44	138.81	22.02	40.66	68.03
	26	C	23	22	17	1.52	109.80	165.74	19.83	38.26	67.37
	27	C	26	23	22	1.52	112.85	− 56.83	19.28	36.90	67.75
	28	C	27	26	23	1.51	112.96	− 173.97	18.92	36.05	66.55
dHbS (2hbs)	8	N	3	2	1	1.35	118.60	146.55	25.47	30.51	51.74
	9	C*	8	3	2	1.47	121.18	− 179.95	26.82	31.08	51.59
	10	C	9	8	3	1.52	112.95	− 45.56	27.72	30.26	50.67
	11	O	10	9	8	1.22	121.01	141.18	28.49	30.81	49.89
	12	C	9	8	3	1.52	102.59	− 165.60	27.34	31.07	53.03
	13	C	12	9	8	1.48	101.88	− 36.76	26.12	31.40	53.81
	14	C	8	3	2	1.47	128.32	− 1.17	25.06	30.52	53.16
	15	N	10	9	8	1.32	115.69	− 37.42	27.57	28.94	50.74
	16	C*	15	10	9	1.45	122.40	179.51	28.35	28.03	49.92
	17	C	16	15	10	1.52	109.54	− 65.19	27.97	28.22	48.45
	18	O	17	16	15	1.22	120.66	137.59	28.84	28.24	47.58
	19	C	16	15	10	1.54	110.83	172.40	28.11	26.56	50.35
	20	C	19	16	15	1.52	109.82	− 65.36	28.65	26.35	51.76
	21	C	19	16	15	1.53	110.49	55.74	26.61	26.23	50.32
	22	N	17	16	15	1.32	116.39	− 41.69	26.68	28.39	48.18
	23	C*	22	17	16	1.45	121.88	178.45	26.19	28.62	46.83
	24	C	23	22	17	1.53	110.05	− 69.01	26.63	30.00	46.34
	25	O	24	23	22	1.22	120.50	141.81	26.98	30.17	45.18
	26	C	23	22	17	1.53	110.69	169.71	24.67	28.52	46.78
	27	C	26	23	22	1.51	114.06	− 65.70	24.13	27.13	47.08
	28	C	27	26	23	1.51	112.65	− 178.53	22.61	27.06	46.99

C* = Chiral Carbon given in Fig. 2a.

**Table 4:** Peptide bond torsion in PGG and PVG residues.

Tripeptide in β-Chain Link	Residues	dHbA (PDB ID)	OHbA (PDB ID)	dHbS (PDB ID)	OHbS	
					Untreated (PDB ID)	Treated (PDB ID)
Pro–Glu–Glu	Pro–Glu	174.14 (1a3n)	− 174.33 (1hho)			
	Glu–Glu	179.45 (1a3n)	170.93 (1hho)			
Pro–Val–Glu	Pro–Val			179.51 (2hbs)	− 178.88 (5e6e)	179.48 (5e83)
	Val–Glu			178.45 (2hbs)	179.82 (5e6e)	175.67 (5e83)

### The pH effect study and HbS interactions with 2,3-DPG

The pH effect on dHbS polymerization also gave tremendous facts associated with the control or acceleration of polymerization. When the protonation of the salt bridge-forming residues (Glu7 and Lys132) present on the β-chain of dHbS was done computationally, the salt bridge distance between them was found lesser as compared to their deprotonated forms. Therefore, the effect of protonation and deprotonation on the salt bridge was found directly related to the salt bridge distance. It was also found that the functional group of only Lys132 got protonated, while that of Glu7 did not, after the computational protonation. Furthermore, when the structure of the HbS β-chain (1b86^72^) was evaluated to know the effect of 2,3-DPG entry, an additional salt bridge between 2,3-DGP and the ‘Lys residue’ of ASN-LEU-LYS-GLY-THR peptides was noted.

## Discussion

**Figure 4:**
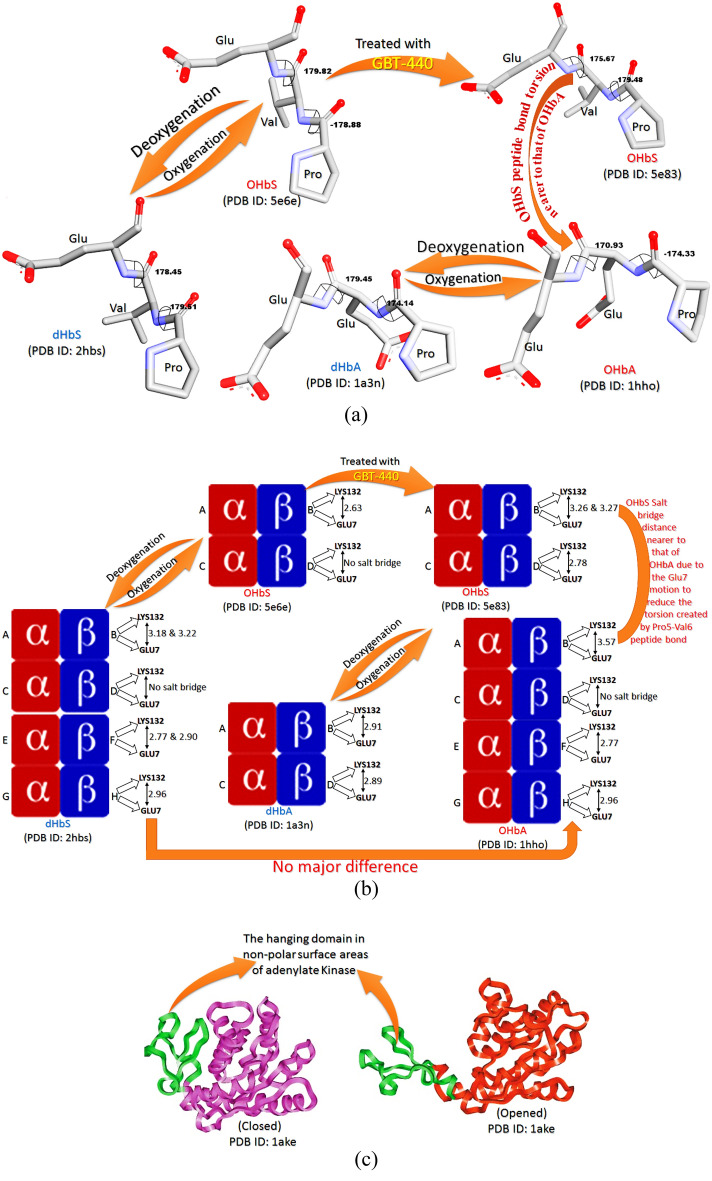
(**a**) OHbS → dHbS conversion involving the distortion in PVG residues torsion, (**b**) biophysical interactions made by PGG/PVG residues of HbA/HbS in different conditions, and (**c**) flexibility found in the hydrophobic domain^73^.

The results interpretation in the presented work removed the curtain from not only the reason for dHbS polymerization but also the mechanism of GBT440 action. In the results of physiochemical parameters among biological molecules (OHbA, dHbA, OHbS, and dHbS), the Gly7-Lys132 salt bridge distance on β-chain (B chain) was found greater in OHbA as compared to dHbA (Tables 1, 2). The short-ranged Glu7–Lys132 salt bridge in dHbA is an indication of its stability which makes the local environment rigid. This is because the stabilizing salt bridges make local regions inflexible for better fit due to functional requirements^20^. The formation of this more stable and short-ranged salt bridge can be related to the change in Pro5–Glu6 peptide bond torsion (Table 4). It is because, the conversion of OHbA to dHbA involves the change in Pro5–Glu6 peptide bond torsion to a greater extent i.e. from − 174.33 to + 174.14, while it occurs only to some extent in Glu6–Glu7 peptide bond i.e. from + 170.93 to + 179.45 (Fig. 4a). Actually, this torsional change occurs due to the motion of Pro5 residue around the Pro5–Glu6 peptide bond as per the Ramachandran plot (Fig. 3a,b). It is because the value of Ф (phi) bond is decreased for Pro5, and that of Ѱ (psi) for the same increased, while only minor changes are observed in values of Ф (phi) and Ѱ (psi) bonds for Glu6–Glu7 (Fig. 3a,b). Hence, during the torsional change in the Pro5–Glu6 peptide bond along with the OHbA conversion to dHbA, the Pro5 motion in the Pro5–Glu6 peptide bond creates a torsion due to which Glu6 moves in the opposite direction with respect to Glu7 (Fig. 4a). In this way, the movement of Glu6 in opposite direction minimizes the created torsion. Besides minimization, the motion of Glu6 in the opposite direction concerning Glu7 also reduces the repulsion between two negatively charged species i.e. Glu6 and Glu7. Because of feeling less repulsion, Glu7 becomes able to make a more stable short-ranged salt bridge with Lys 132 after the conversion of OHbA to dHbA (Fig. 4a,b). In this way, the PGG residues in dHbA get stabilized due to this short-ranged and stronger salt bridge.

On the other hand, the salt bridge distance on the β-chain (B chain) was found lesser in OHbS as compared to dHbS. The long-ranged Glu7–Lys132 salt bridge in dHbS is the indication of its instability due to which the peptide chain feels conformational specificity for the fold or function which is provided by a destabilizing salt bridge^23^. Moreover, the increase in salt bridge distance in the case of dHbS is not only an indication of a weaker salt bridge but also responsible for the conversion of PVG into a movable/moving domain. This is because short-range electrostatic interactions are largely absent between moving domains^73^. Therefore, the Pro–Val–Glu residues gain flexibility in dHbS. Another reason behind the flexibility of Pro–Val–Glu is the hydrophobic nature of the ‘valine’ residue present in Pro–Val–Glu of HbS. It is because not only the weaker electrostatic interactions (salt bridge) but also the hydrophobicity allows conformational flexibilities in a protein^14,73^ as shown in Fig. 4c. Moreover, the torsional change in Pro–Val–Glu peptide bonds along with the OHbS conversion to dHbS can not be ignored. The observation of the negatively charged Glu7 motion away from the charged Lys132 residue was done with the inclusion of a Ramachandran plot-based study (Fig. 3c,d). Besides, it also played an important role in the evaluation of all the things mentioned in the introductory part of the presented article. Actually, the conversion of OHbS to dHbS involves the change in Pro5–Val6 peptide bond torsion to a greater extent i.e. from − 178.33 to + 178.14 (Fig. 4a) which was found more as compared to Pro5–Glu6 in HbA. It is just because of the less hindered Pro5–Val6 peptide bond which makes Pro5–Val6 more movable around the peptide bond in HbS as compared to Pro5–Glu6 in HbA. Hence, the motion in Pro5–Val6 residues to a greater extent along with the OHbS conversion to dHbS creates a torsion on the Val6–Glu7 peptide which is minimized by the Glu7 motion. For this minimization, the Glu7 residue of HbS moves in that direction where the effect of torsion created by Pro5–Val6 peptide on Val6–Glu7 peptide could be minimal. This notable point regarding the Glu7 motion was also supported by the Ramachandran plot-based study (Fig. 3c,d) in which, the value of Ѱ (psi) bond for Glu7 was found to increase, and that of Ф (phi) for the same was found with no major change, while only minor changes were observed in values of Ф (phi) and Ѱ (psi) bonds for Pro5 and Val6. Subsequently, due to the occurrence of Val6–Glu7 peptide motion in the opposite way with respect to Pro5–Val6 peptide (Fig. 4a), a less stable salt bridge with a long distance is made between Glu7 and Lys132 after the OHbS conversion to dHbS (Fig. 4b). Thus, the PVG residues in the deoxygenated form of HbS get destabilized due to the long-ranged and weaker salt bridge. In this way, it clearly clarifies how the Val6 in place of Glu6 does not involve directly in dHbS disability as discussed in the section “Biophysical interaction-based evaluation”

The use of the anti-sickling drug (GBT440) establishes two salt bridges (Table 2) between Glu7 and Lys132 (Fig. 4b), so that the motion of PVG can be stopped, and the HbS structure can be stabilised. Because of this, the conversion of PVG into a moving domain is also stopped. Besides, in GBT440 treated OHbS (5e83^13^), the additional salt bridge (B:LYS82:NZ-D:HIS146:O) formed between two different β-chains (Table 2) was also found responsible for OHbS stability. It also exhibits how this anti-sickling drug (GBT440) makes OHbS stable by increasing the number of salt bridges. If it is so, all these observed things should be done by another anti-sickling drug, VZHE-039. When the same things were studied in the case of VZHE‑039, it was observed that the VZHE-039 also establishes two salt bridges between the same residues where GBT440 does, but of a longer distance. In VZHE-039 treated HbS, the absence of additional salt bridges and the presence of long-ranged salt bridges indicate the lower ability of VZHE-039 to make the HbS structure stable as compared to GBT-440.

Moreover, the use of the anti-sickling drug (GBT440) also decreases the torsion in the Val6–Glu7 peptide bond to some extent but increases in the Pro5–Val6 peptide bond residue to a greater extent (Fig. 4a). In GBT440 treated OHbS, the Val6–Glu7 peptide torsion was found nearer to the torsion of Glu6–Glu7 peptide in OHbA (Fig. 4a) which evidently shows that the further torsion in Val6–Glu7 peptide to make the Glu7 movable is very difficult. Through this restriction in further torsion at the mutation point (PVG), the structure of OHbS becomes more stable when treated with GBT440. Furthermore, due to this torsional restriction on Val6–Glu7 peptide via GBT440, HbS-PVG attains a similar orientation as HbA-PGG attains, and the Glu7–Lys132 salt bridge distance in OHbS also becomes equal to OHbA (Fig. 4b). Consequently, the conversion of stable OHbS into less stable dHbS is stopped by GBT440. In this way, the anti-sickling drug (GBT440) stops its participation in the polymerization reaction by making the oxygenated form of HbS more stable via additional salt bridges and restriction in PVG peptide bond torsion.

Another reason for the same thing is the hydrophobic nature of Pro–Val–Glu peptides because these peptides have two hydrophobic residues (Pro and Val) and one hydrophilic residue (Glu). On the other hand, Pro–Glu–Glu peptides has two hydrophilic residues (Glu) and one hydrophobic residue (Pro). Actually, the presence of two hydrophobic amino acids in Pro–Val–Glu peptides them hydrophobic. On the other hand, the binding sites i.e. β88 leucine (Leu-beta88) and β85phenylalanine (Phe-beta85) residues on the adjacent dHbS-M are also hydrophobic^74^. Hence, the attachment of hydrophobic Pro–Val–Glu of HbS with the hydrophobic residues of the adjacent dHbS-M is stronger as compared to that of hydrophilic Pro–Glu–Glu of HbA. It is because hydrophilic compound does not have a greater affinity towards hydrophobic complex, but hydrophobic compound does, as per universal truth.

### Key phases before RBC sickling

**Figure 5:**
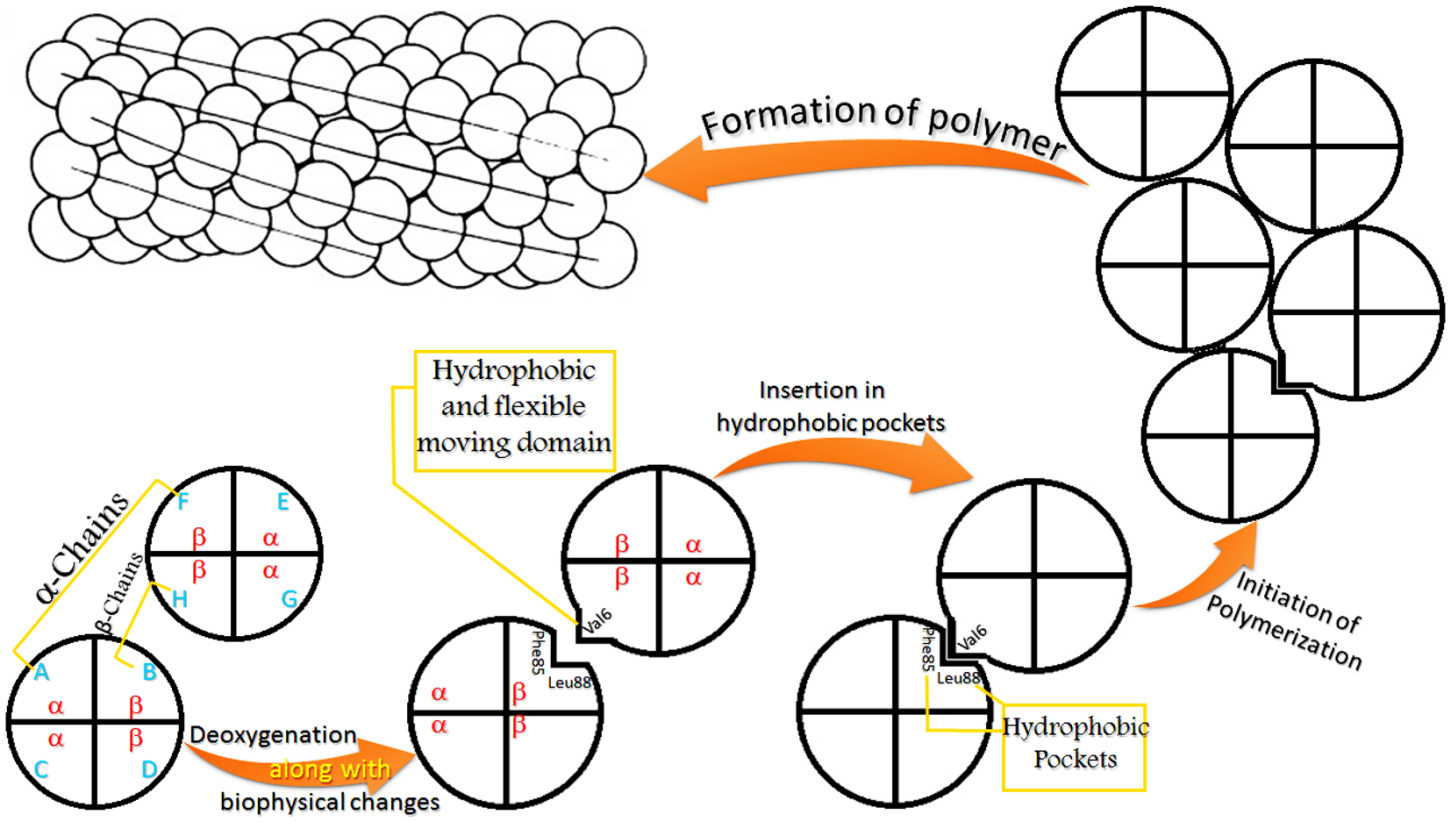
HbS polymerization mechanism after deoxygenation along with biophysical changes^105^.

After a deep computational evaluation of RBC sickling-related data, the steps involved before the polymer formation are summarized below (Fig. 5):

- Deoxygenation of HbS^5,13^
- Increase in Glu7-Lys132 salt bridge distance (Fig. 4b) along with the torsional change in peptide bond (Fig. 4a) as per the literature data^7,75^.
- Destabilization of Glu7-Lys132 salt bridge due to the increased distance^23^
- Conformational specificity for the fold or function provided by a destabilized salt bridge^14,76^
- Creation of the moving domain through flexibility^73^ caused by hydrophobic nature^14^
- Insertion of the hydrophobic moving domain into hydrophobic pockets on adjacent dHbS-M^30,34^.
- Initiation of HbS polymerization for the sickling of RBC.

### GBT-440 anti-sickling mechanism

After a deep evaluation of the pdb file with an anti-sickling drug (GBT-440), the mechanism of GBT-440’s action to stop the polymerization is summarized below:

- Accumulation of GBT-440 in oxygenated HbS
- After accumulation, the condensation reaction between αVal1 N-terminal amines and GBT440 for the formation of Schiff‘s base.
- Variation in torsional strain (Fig. 4a) on peptide bonds after the Schiff‘s base formation.
- The motion of the salt bridge-forming residues toward other ionic residues (Fig. 4a) for torsional strain minimization.
- Creation of the additional salt bridges (5e83^13^) in the HbS structure (Table 2) through the motion of ionic residues to each other (Fig. 4a).
- Establishment of stable OHbS from untreated OHbS due to the formation of additional salt bridges (Fig. 4b) and Table 2.
- After gaining stability, neither the creation of the hydrophobic moving domain in HbS to be inserted in dHbS-M nor HbS polymerization for RBC sickling.

The effect of carbon monoxide combination with HbA was observed by studying the variation in interactions among polypeptide residues. The increased number of salt bridges in HbA after the addition of CO openly indicates its stability. Hence, HbA gets stability through the increased number in the salt bridge after combination with CO, while no salt bridge increment was observed in HbA oxygenated with oxygen gas (Table 1). Due to this, HbA combines with CO more tightly as compared to O_2_. Besides, no changes occur in the structure of CO, but that in HbA occurs after a combination of both. Therefore, in HbA and CO combination, HbA has a greater affinity for the same, but CO does not.

**Figure 6:**
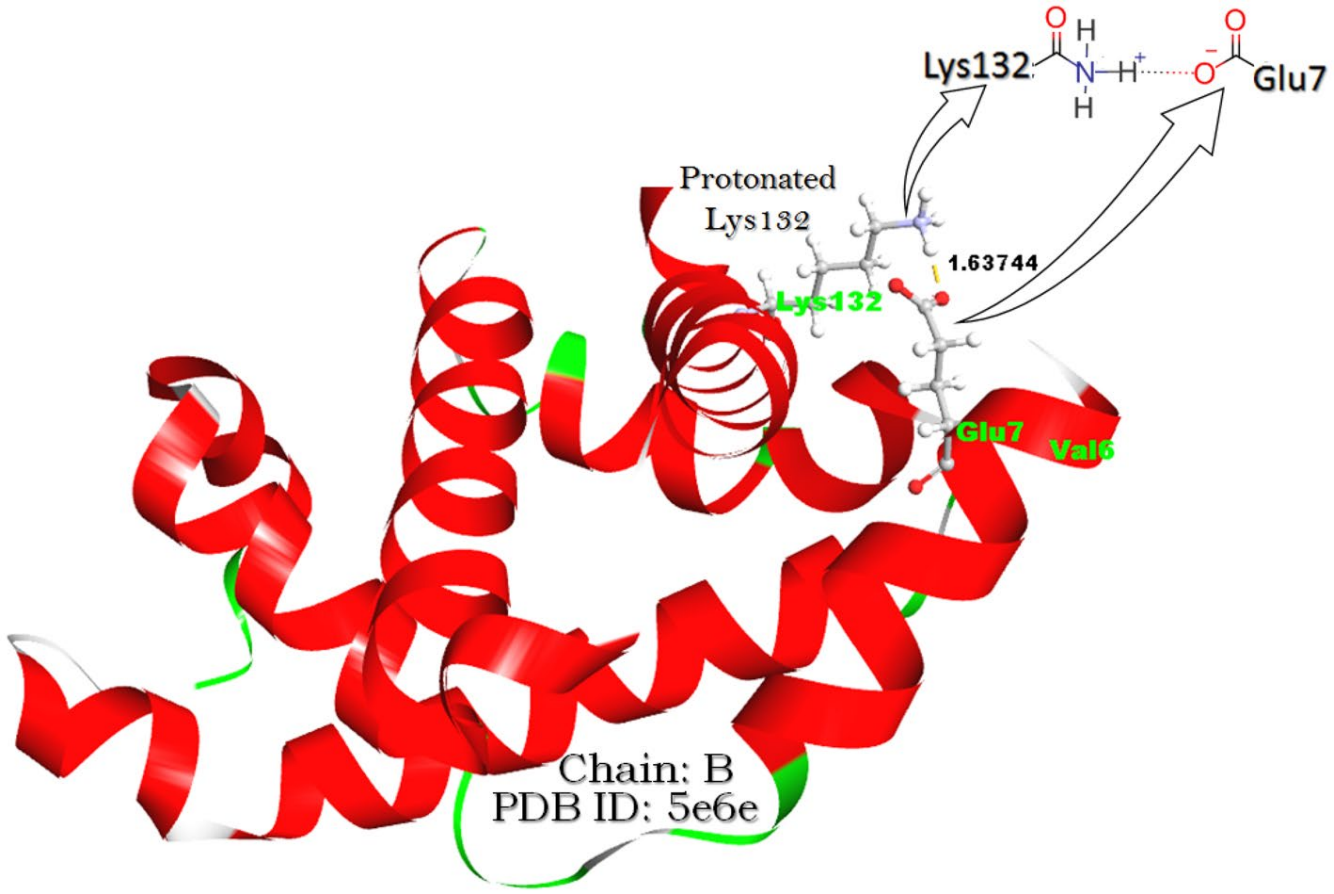
The Glu7–Lys132 salt bridge distance in OHbS after computational protonation.

During the study of the pH effect on dHbS polymerization, it was found that the protonation of only Lys132 was just because of the lone pair donor atom in Lys132 i.e. nitrogen atom of the amine group (–NH_2_) of Lys132 (Fig. 6). The nitrogen atom of the amine group (–NH_2_) makes a coordinate bond with the H^**+**^ ion to convert into its protonated form (–NH_3_^**+**^). On the other hand, the deprotonated form of Glu7 has a carboxylate ion (Fig. 6) which is considered more stable due to the presence of delocalized electrons^77^. Hence, the protonation of Glu7 does not happen to maintain the stability of Glu7. Moreover, the decreased salt bridge distance was just because of the existence of H^**+**^ ion between Glu7 and Lys132 due to which Glu7 becomes unable to make a salt bridge with Lys132 directly (Fig. 6). In this way, the ionic strength of Lys132 residue to make HbS structure stable is diminished because the protonation of the polymeric chain makes the ionic strength weaker^78^. In the same way, the protonation also makes the polypeptide chain weaker because as per literature data^78^, the protonation involves breaking intra-chain hydrogen bonds. Based on this information, it can be pointed out that not only the dHbS instability but also the instability of the Glu7–Lys132 salt bridge makes the local environment flexible. This is because the destabilizing salt bridges make local regions flexible for better fit due to functional requirements^20^. Notably, the effective folding rates of the Lys-based peptides at acidic pH are slightly slower^75^ which also shows that the unfolding in the HbS protein chain becomes very easy for the conversion of a peptide chain into a moving domain.

**Figure 7:**
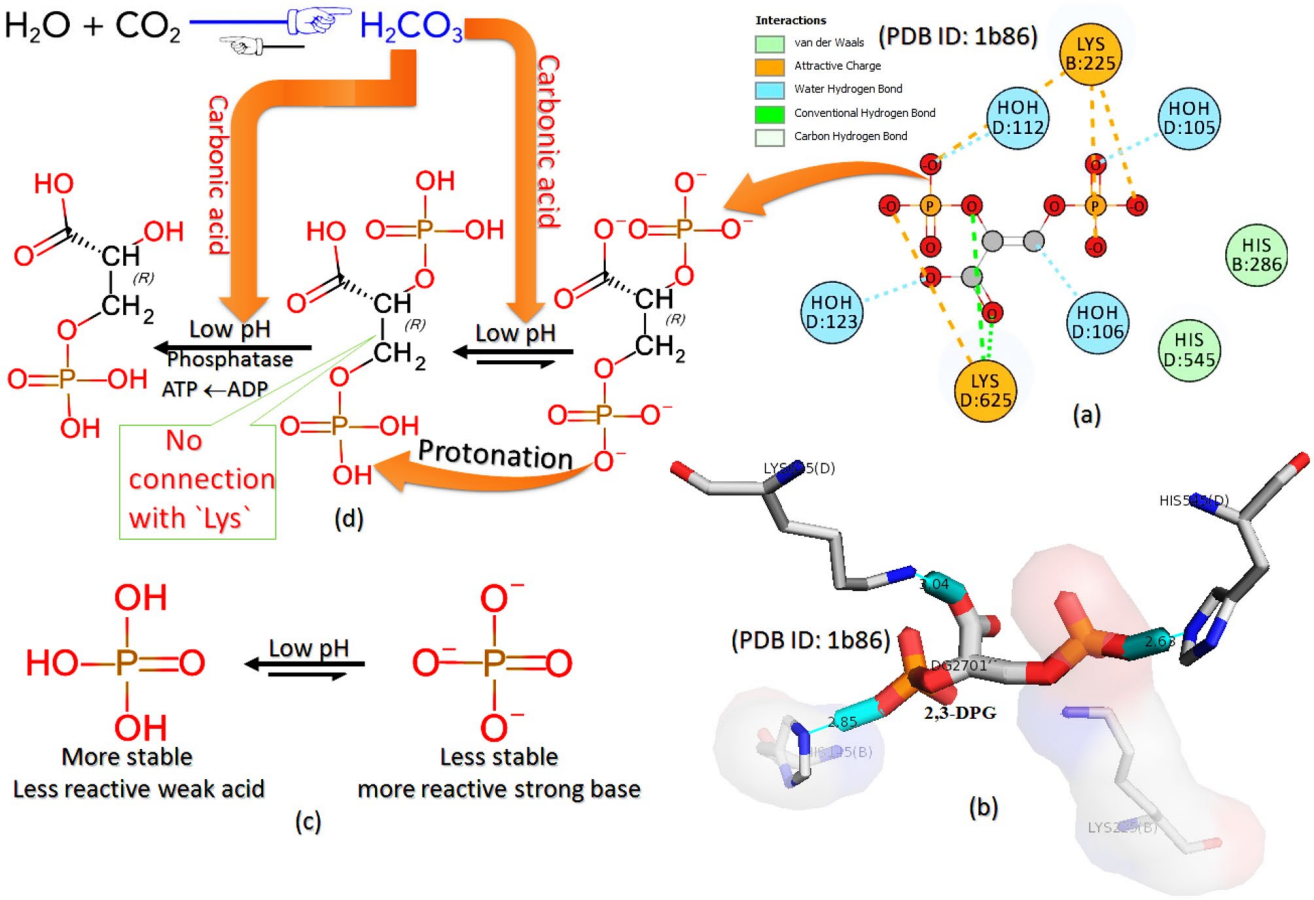
(**a**) 2D and (**b**) 3D view of the 2,3-DPG interactions with ‘Lys’ (**c**) phosphate and phosphoric interconversion and (**d**) the mechanism of 2,3-DPG conversion in 3-phosphoglycerate.

Additionally, when the entry effect of 2,3-DPG and GBT440 in the hemoglobin structure was analyzed, it was noted that the effect of 2,3-DPG entry in hemoglobin structure (1b86^72^) was found just conflict with that of GBT440 (5e83^13^). The 2,3-DPG makes a salt bridge (Fig. 7a,b) with ‘Lys residue’ of ASN-LEU-LYS-GLY-THR peptides present in the hemoglobin β-chain (1b86^72^). Interestingly, the same ‘Lys residue’ was found attached to the ‘His residue’ via an additional salt bridge (B:LYS82:NZ-D:HIS146:O) (5e83^13^) in the GBT440-treated HbS structure. Hence, it can be concluded that the presence of GBT440 allows the ‘Lys residue’ to make the salt bridge with the ‘His residue’, but that of 2,3-DPG does not. Moreover, the most important fact regarding the additional salt bridge (B:LYS82:NZ-D:HIS146:O) in GBT440-treated HbS, was its existence between two different β-chains (B and D) because both residues were found on different β-chains (5e83^13^). It confirms the stability of GBT440-treated HbS in which the two different β-chains are connected via B:LYS82:NZ-D:HIS146:O in which the ‘Lys residue’ of the ‘B chain’ interacts with another ionic residue of the ‘D chain’ (Table 2). Therefore, it is clear that the GBT440 increases the number of salt bridges in HbS (Fig. 4b) to make the HbS protein more stable. On the other hand, the binding of 2,3-DPG in the β-cleft promotes the polymerization of deoxy-HbS^10^. It is because the 2,3-DPG does not let the Lys residue of ASN–LEU–LYS–GLY–THR peptides to make a salt bridge with ‘His residue’ as GBT440 does. Due to this, the hemoglobin structure with 2,3-DPG becomes less stable. In this way, we can say that the 2,3-DPG creates a disturbance via salt bridge formation in the hemoglobin structure and makes it less stable. This destabilization is managed in HbA properly^42^ but not in HbS as per the presented study. Hence, the 2,3-DPG entry in HbA is not found as dangerous as in HbS.

The entry/removal of 2,3-DPG is associated with pH variation. The low pH is responsible for the removal of 2,3-DPG, while the higher pH is responsible for the entry of 2,3-DPG as per the introductory part. Actually, it is just because of the presence of two phosphate (PO_4_^3^) groups and one carboxylate group in the 2,3-DPG structure (Fig. 7a,b) which are less stable but strong bases as they are produced from their respective stable acids i.e. phosphoric acid (H_3_PO_4_) and carboxylic acid (–COOH). It can be explained by keeping the facts collected after getting literature data. For instance, we know that the phosphoric acid (H_3_PO_4_) is a weak and stable acid^79,80^, so the phosphate ion (PO_4_^3**−**^) must be a strong base with less stability according to the collected works (https://www.chem.fsu.edu/chemlab/Mastering/PhosphateBuffers.htm) because a base of a chemical species will be strong if its protonated form is classified as a weak^81^. Therefore, the conversion of phosphoric acid to phosphate is a very slow process but that of phosphate to phosphoric acid is very fast (Fig. 7c). Consequently, the affinity of less stable phosphate ion (PO_4_^3−^) for H^**+**^ or protonation is found more to get stabilized form. Besides, the presence of two phosphate (PO_4_^3**−**^) ions in the 2,3-DPG structure (Fig. 7a) is also found responsible for the formation of a salt bridge with ‘Lys residue’. It is because the salt bridge is formed between two oppositely charged species^21^ for which the phosphate group (PO_4_^3**−**^) has a negative charge while ‘Lys residue’ has a positive charge. On decreasing pH or increasing H^**+**^ ion concentration, the protonation of two negatively charged phosphate (PO_4_^3**−**^) ions takes place (Fig. 7d). Such type of protonation neutralizes the negatively charged phosphate^82^ to convert into their protonated and neutral form (Fig. 7d). In this way, after loosing its negative charge, the 2,3-DPG becomes unable to make the salt bridge with positively charged ‘Lys residue’, and conditions favors the removal of 2,3-DPG from HbS structure. Hence, the deprotonated 2,3-DPG conversion into protonated 2,3-DPG was found responsible for not only 2,3-DPG less stability but also its removal from the intracellular environment of low pH (Fig. 7d). Among all these described things, the involvement of phosphatase activation at low pH can not be ignored. Phosphatase removes the phosphate group from the organic residue^83^. The activation of phosphatase is also found responsible for the removal of 2,3-DPG from intracellular medium^84^. The medium of the intracellular environment is made acidic by the addition of CO_2._ It is because carbon dioxide, already acidic in nature^85^, forms carbonic acid (Fig. 7d) after the reaction with water^86–88^. Hence, the higher concentration of CO_2_ means more production of carbonic acid which makes the intracellular medium acidic by lowering pH (7–5.6)^89^. Along with lowering the pH value, the phosphatase becomes active since the phosphatase is inactive at pH levels above 7.2 but is more active at the lower pH in RBCs^90^. Hence, after activation at low pH, the phosphatase starts to cleave the ester bond formed between the phosphate group and organic residue i.e. glyceric acid (Fig. 7d). At low pH, the cleavage of phosphate groups becomes easier due to the lack of 2,3-DPG connection with ‘Lys residue’ as described above. In this way, the phosphatase activation contributes to the decreased 2,3-DPG levels^90^ by converting it into 3-phosphoglycerate^91,92^ (Fig. 7d). Therefore, in the presented study, the lower pH was found responsible for not only the weaker/unstable 2,3-DPG connection with ‘Lys residue’ but also the phosphatase activation. On the other hand, all these things happen oppositely at higher pH which was found responsible for not only 2,3-DPG stability but also its entry in the intracellular environment.

Henceforward, the same mechanism of 2,3-DPG to cause trouble in hemoglobin can be applied in the HbS-typed hemoglobin before polymerization. It is because the effect created by the entry of 2,3-DPG in normal hemoglobin was found very adverse in sickle hemoglobin as the 2,3-DPG entry helps in the oxygen release from HbA^93^ i.e. deoxygenation of HbA. The deoxygenated form of HbA can get stabilized but that of HbS is not, as described above, that is why dHbS undergoes polymerization. Keeping the described facts into consideration, the depletion of 2,3-DPG could also play a key role in the control of HbS polymerization, which has been suggested recently^44^.

**Figure 8:**
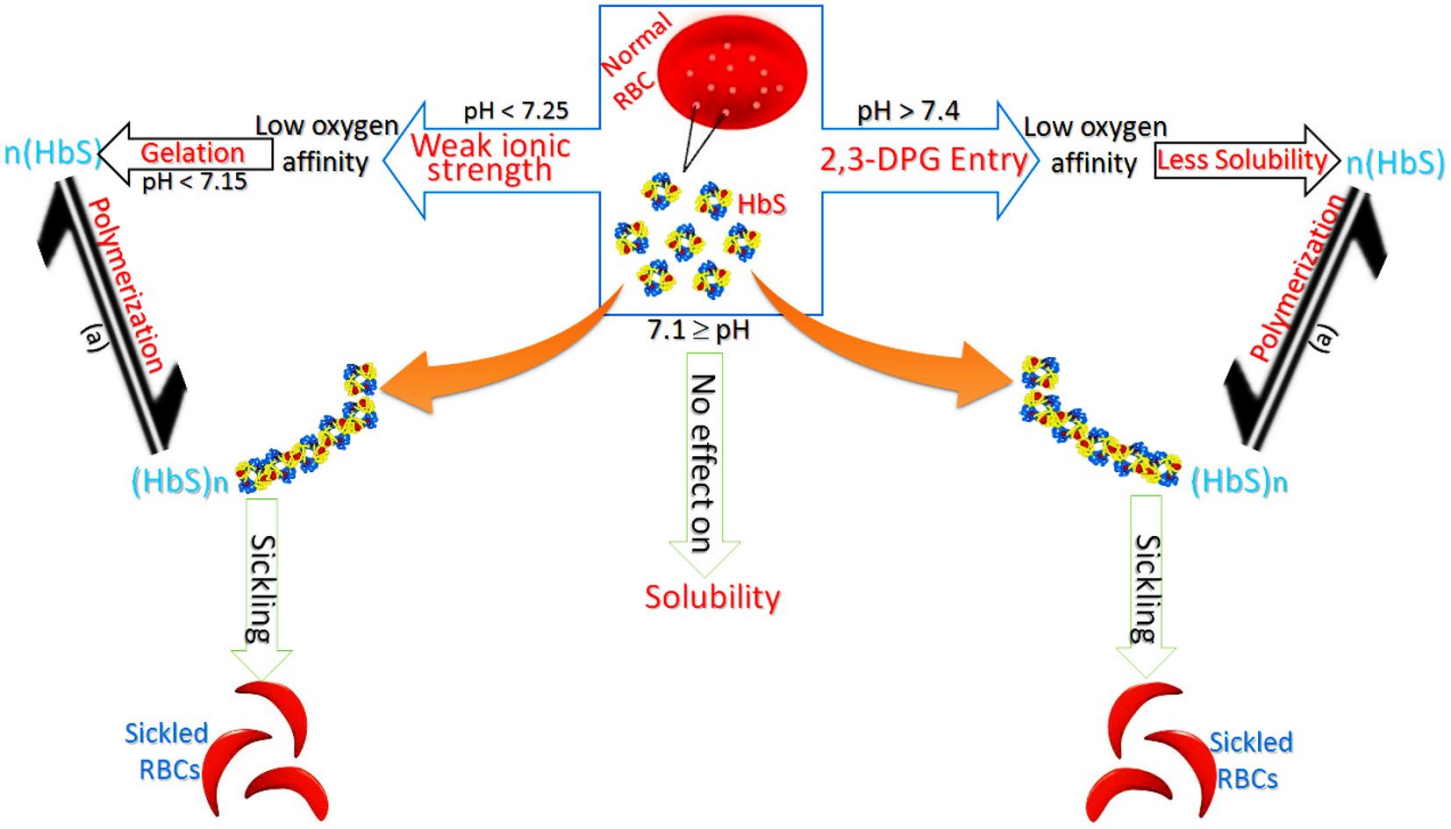
The pH effect on the solubility, gelation, binding affinity for oxygen, and polymerization of HbS; (a)^66,106^.

After a deep evaluation of RBC sickling-based literature data, a symbiotic effect of pH and 2,3-DPG on the dHbS solubility was observed. Besides, the pH effect seemed just like a mysterious fact that was found associated with dHbS polymerization before RBCs sickling. Truly, RBC sickling can occur in both conditions i.e. whether the pH of the intercellular medium is increased^39^ or decreased^39,95^. At higher pH (more than 7.4), the HbS polymerization occurs due to the decreased solubility caused by 2,3-DPG accumulation^39^ (Fig. 8), while at lower pH (less than 7.25), the HbS polymerization can also take place just because of HbS gelation^39^ (Fig. 8). It is because the increase in pH promotes the accumulation of 2,3-DPG that causes a large release of oxygen from red cells, whose risk of sickling is high^39^. Moreover, the intracellular pH was found 7.41 in the case of HbS deoxygenation^10^. It shows that the higher pH value of the intracellular medium favors the release of oxygen from HbS caused by 2,3-DPG accumulation. On the other hand, the decrease in pH causes gelation which is also found responsible for a large release of oxygen from red cells, whose risk of sickling is high^39^. Moreover, the intracellular pH was found 7.13 in the case of HbS oxygenation^10^. At this pH, the HbS gelation was confirmed when a sharp increase in the gelling tendency was observed below the pH 7.15^39,94^. We know that gelation is a general way to convert a fluid into a solid^96^. Hence, the gelation increases the density of a substance^96^. The same things can be applied to the HbS gelation at low pH, which occurs due to the protonation of the HbS polypeptide chain because the protonation not only makes a polymeric chain denser but also provides stiffness to it^78^. It has already been confirmed that denser HbS cells also cause low oxygen affinity, and increase the risk of RBC sickling^97^. Moreover, the protonation diminishes the ionic strength of a polymeric chain^78^; which clearly shows the HbS disability via weak ion strength (salt bridge) at low pH. In this way, we can say that the protonation of the HbS polypeptide chain occurs in an acidic medium continuously until its ionic strength diminishes. The completion of protonation of the HbS polypeptide chain may occur at pH 7.15 after which HbS gelation starts^39^, and then polymerization occurs for RBC sickling. It is because no pH effect was observed on Hb and Hb-DPG solubility^98^ below 7.15 pH or neutral pH (Fig. 8) i.e. no more consumption of proton (H^**+**^) as all residues get protonated completely at pH 7.15. The same results were observed below pH 7 when only a slight drop in the maximum number of sickled cells^39^ was noted. It openly revealed no need for protonation as all residues get protonated at pH 7.15.

## Conclusion

Based on the interpretations of the results, it can be concluded that many factors were found associated with HbS polymerization after deoxygenation. In these factors, peptide bond torsion, salt bridge number, salt bridge strength, and hydrophobicity can be included. In this way, we can say that an increase in the HbS oxygen affinity; HbS solubility; and HbS stability through the increased salt bridge number could stop HbS polymerization easily. In addition, the intracellular elimination of 2,3-DPG and pH maintenance can also do the same thing. Of course, the separate effects of 2,3-diphosphoglycerate and intracellular pH on HbS were also observed, but they were found to show symbiotic effects on the dHbS solubility at equilibrium that were shown responsible for HbS polymerization in RBCs.

Moreover, the mechanism and strength of the antisickling drugs other than GBT440 can also be evaluated by following the presented method. It can also be concluded that the antisickling drug that forms the Schiff base with ‘Val residue’ of α-chain terminal would behave like GBT440 or not, is not confirmed. For the evaluation of its antisickling properties, we have to study its accumulation effect in the HbS structure including the salt bridge number, peptide bond torsion, and other factors as described above. By doing so, it would become very easy to know how the newly synthesized drug during its antisickling activity makes the HbS structure not only stable but also unconvertable in the moving domain.

## Investigational

### Software and tools

All the software and tools used for the current study are Discovery Studio Visualizer 2019 (v19.1.0.18287)^99^, PyMOL, GaussView 6.0^100^, Marvin Sketch (16.9.12 version), and Protein Data Bank (PDB).

### Simulation study

In the presented computational study, the pdb files of oxygenated normal hemoglobin (OHbA), deoxygenated normal hemoglobin (dHbA), oxygenated sickle hemoglobin (OHbS), and deoxygenated sickle hemoglobin (dHbS) with pdb codes 1hho^67^, 1a3n^68^, 5e6e^69^ and 2hbs^70^ respectively, were obtained from the protein data bank. After that, all significant physiochemical parameters of all obtained files were studied and compared deeply. Besides the taken pdb files^67–70^, the most significant data of other pdb files such as 5e83^13^, 6xd9^53^, 2m6z^71^, and 1b86^72^ was also included in the current study. The pdb file with pdb code 5e83^13^ was taken for the structural study of OHbS having an anti-sickling drug, GBT440 so that its mechanism to make the HbS structure stable and to stop the HbS polymerization, could be explained. On the other hand, a 6xd9^53^ pdb coded file was used for a comparative study purpose so that the comparison between GBT440 and VZHE-039 could be done to evaluate their abilities to make the HbS structure stable. It is because the drug named VZHE‑039 has also been suggested as a candidate to stop RBCs sickling. The pdb file with pdb code 2m6z^71^ was taken to know the effect of carbon monoxide on HbS, while the effect of 2,3-diphosphoglycerate (2,3-DPG) entry in hemoglobin structure was analyzed by the evaluation of pdb file with pdb code 1b86^72^. The main purpose of the presented computational study was to answer of different aspects mentioned in the introductory part through the deep analysis of all Hb-related structures. Some of them are as follows:

### Physio-chemical interaction study

**Figure 9:**
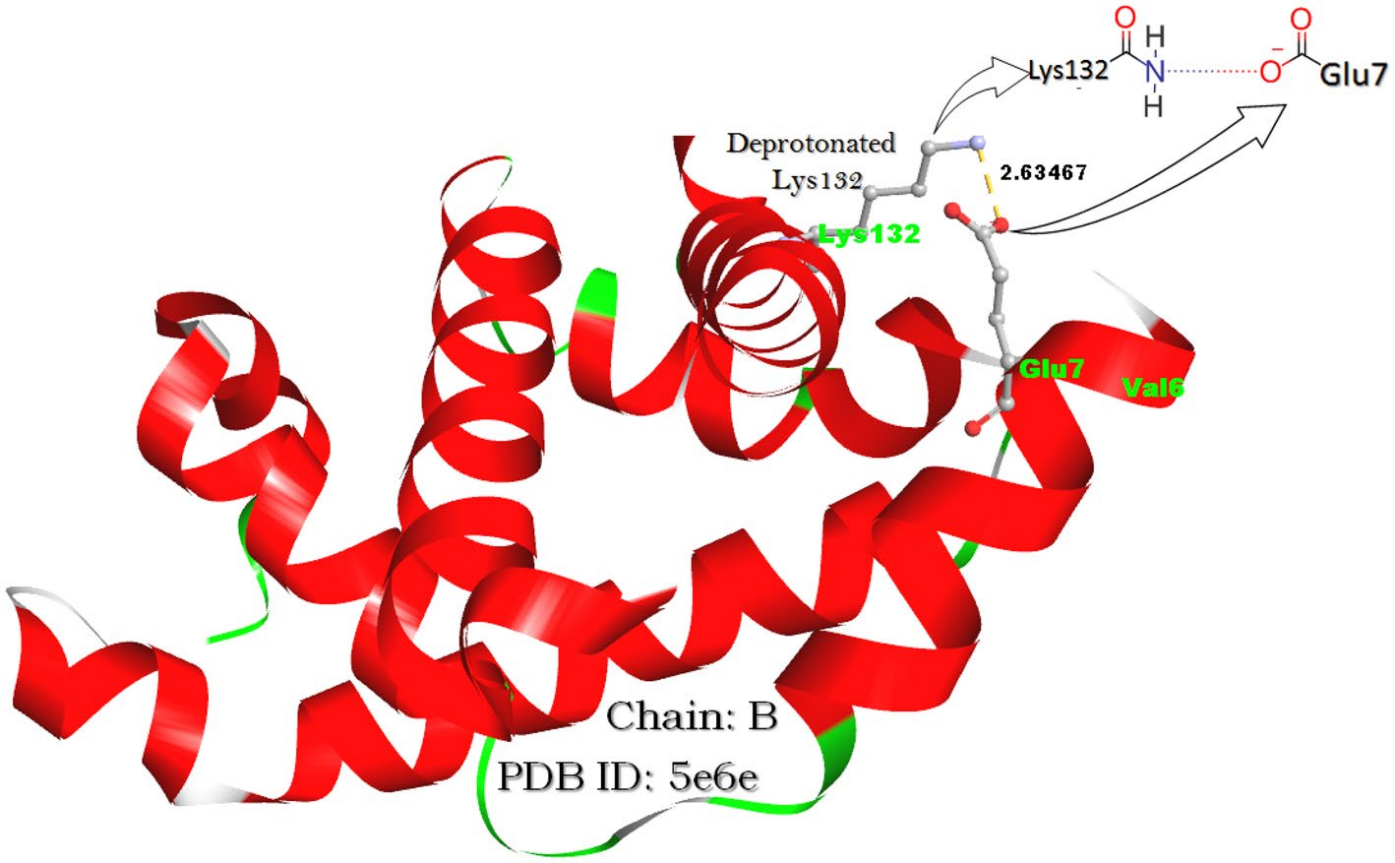
The salt bridge distance between deprotonated Glu7 and Lys132 in OHbS.

For the study of physio-chemical interaction-based differences among biological molecules (OHbA, dHbA, OHbS, and dHbS), all their pdb files were opened in Discovery Studio 2019, and all types of physio-chemical interactions such as salt bridge (Fig. 9), hydrogen bond, and charge-dipole interaction were noted and used for the result interpretation. The salt bridge interactions are relatively strong non-bonded interactions between pairs of oppositely charged groups where hydrogen bonding also occurs. To know the mechanism of the anti-sickling drug action, the analysis of electrostatic interactions, especially salt bridges number in GBT440 treated OHbS (5e83^13^) was done by comparing its β-chain with those of OHbA, dHbA, OHbS, and dHbS having pdb codes: 1hho^67^, 1a3n^68^, 5e6e^69^ and 2hbs^70^ respectively.

Besides, the peptide bond torsion was also studied among the same. It is because amino acids in a protein chain feel torsion strain^101–103^ which can cause a resistance to bond rotation and can influence a barrier to rotation^103^**.** Hence, the Ramachandran plot-based study was also done to evaluate the change in peptide bond torsion occurring in Pro–Glu–Glu as well as Pro–Val–Glu along with the OHbA/OHbS conversion into dHbA/dHbS. The inclusion of the Ramachandran plot-based observations was found very supportive in the result interpretation of the presented study. All these similar things were done with GBT440-containing OHbS to know how this anti-sickling drug affects the peptide bond torsion in HbS structure, and how it makes the behavior of OHbS equal to OHbA. In the study of physiochemical differences among biological molecules (OHbA, dHbA, OHbS, and dHbS), the result interpretations gave many key points. Based on these significant points, it became very easy to explain the reason for dHbS polymerization before RBCs sickling. The additional literature data related to the taken biological molecules was also included in the result interpretation to support the main find findings of the physiochemical study. The main purpose of the presented study was to evaluate different parameters changed during the OHbS conversion to dHbS. Here, the scanty data related to the changes occurring in HbA during the same were also evaluated for a comparative study between HbA and HbS. Due to this, the torsion angles associated with Pro–Val and Val–Glu peptide bonds before and after deoxygenation in the HbS protein were studied genuinely but not in the HbA protein. For this purpose, phi (φ) was the Ca(i-1), C(i), N(i), Ca(i) torsion angle in the Pro–Val peptide bond in which Cα (i-1), C(i), N(i), Cα(i) were tagged as C9, N15, C10, C16 respectively (Fig. 2a,b), while psi (ψ) was the Ca(i), C(i), N(i + 1), Ca(i + 1) torsion angle in Val–Glu in which Cα(i), C(i), N(i + 1), Cα(i + 1) were tagged as C16, C17, N22, C23 (Fig. 2a,b). An amino acid needs to be bonded to two other amino acids to have values set for both psi and phi, for that reason, the two terminal amino acids (Thr and Lys) do not have values set for these angles.

### The study of pH, CO, CO_2,_ and 2,3- DPG effects on HbS

Of course, many studies^39^ have shown the dependency of dHbS polymerization on pH. As per the introductory part of the presented article^7,39^, the dHbS polymerization is accelerated by an acidic medium. It is well understood that the acidic medium has a large number of H^**+**^ ions. Due to this, many residues present in the peptide chain get protonated. In the presented study, only salt bridge-forming residues present in PVG and PGG were protonated computationally i.e. Glu7. Besides Glu7, the residue ‘Lys132’ was also protonated as it was found attached to Glu7 via salt bridge formation. It is because the salt bridge is related to not only the stability of the peptide chain^19–21^ but also the folding of a polypeptide chain^36,37^. In the presented work, after getting the protonated form of the salt-forming residues, the salt bridge strength with respect to its connection between Glu7 and Lys132 was studied so that the acidic medium-based facts could be evaluated deeply. In the same way, the effect of CO_2_ in HbS structure was studied computationally, while the effect of 2,3-DPG on HbS physiochemical parameters in pdb files with PDB Codes 1b86^72^ was also studied. The PDB file with pdb code 2m6z^71^ was evaluated to know the effect of CO. It is because the hemoglobin structure gets stability after combining with CO which is why it combines with CO more tightly but with O_2_, loosely^104^. After combination, the interaction-based stability caused by CO addition in hemoglobin was not found as satisfactory as expected. Hence, the reason for the interaction-based stability of hemoglobin caused by CO is yet to be explained.

## Data Availability

The computational data used in the presented study is publicly available. All pdb files of peptide chains related to HbA and HbS can be obtained from the protein data bank to check the reproducibility of the presented article. For the sake of reader, the direct link and accession numbers are provided below: RCSB PDB - 1HHO: STRUCTURE OF HUMAN OXYHAEMOGLOBIN AT 2.1 ANGSTROMS RESOLUTION. RCSB PDB - 2M6Z: Refined solution structure of Human Adult Hemoglobin in the Carbonmonoxy Form. RCSB PDB - 1A3N: DEOXY HUMAN HEMOGLOBIN. RCSB PDB - 5E6E: Crystal Structure of Carbonmonoxy Sickle Hemoglobin in R-State Conformation. RCSB PDB - 5E83: CRYSTAL STRUCTURE OF CARBONMONOXY HEMOGLOBIN S (LIGANDED SICKLE CELL HEMOGLOBIN) COMPLEXED WITH GBT440, CO-CRYSTALLIZATION EXPERIMENT. RCSB PDB - 6XD9: Carbonmonoxy hemoglobin in complex with the antisickling agent 2-hydroxy-6-((6-(hydroxymethyl)pyridin-2-yl)methoxy)benzaldehyde (VZHE039). RCSB PDB - 2HBS: THE HIGH RESOLUTION CRYSTAL STRUCTURE OF DEOXYHEMOGLOBIN S

## References

[1] Pauling L, Itano HA, Singer SJ, Wells IC (1949). Sickle cell anemia, a molecular disease. Science.

[2] Ingram VM (1957). Gene mutations in human hæmoglobin: The chemical difference between normal and sickle cell hæmoglobin. Nature.

[3] Becklake MR, Griffiths SB, McGregor M, Goldman HI, Schreve JP (1955). Oxygen dissociation curves in sickle cell anemia and in subjects with the sickle cell trait. J. Clin. Investig..

[4] Abdu A, Gómez-Márquez J, Aldrich TK (2008). The oxygen affinity of sickle hemoglobin. Respir. Physiol. Neurobiol..

[5] May A, Huehns ER (1972). The mechanism of the low oxygen affinity of red cells in sickle cell disease. Hamatol. Bluttransfus..

[6] Fabry ME, Desrosiers L, Suzuka SM (2001). Direct intracellular measurement of deoxygenated hemoglobin S solubility. Blood.

[7] Papageorgiou DP (2018). Simultaneous polymerization and adhesion under hypoxia in sickle cell disease. Proc. Natl. Acad. Sci..

[8] Gill SJ (1978). Aggregation effects on oxygen binding of sickle cell hemoglobin. Science.

[9] Kim-Shapiro DB, Noguchi CT, Schechter AN, Gladwin MT, Kato GJ, Novelli EM (2021). Sickle hemoglobin polymerization. Sickle Cell Disease.

[10] Poillon WN, Kim BC (1990). 2,3-Diphosphoglycerate and intracellular pH as interdependent determinants of the physiologic solubility of deoxyhemoglobin S. Blood.

[11] Hutchaleelaha A, Patel M, Silva A, Oksenberg D, Metcalf B (2015). GBT440 demonstrates high specificity for red blood cells in nonclinical species. Blood.

[12] Metcalf B (2017). Discovery of GBT440, an orally bioavailable R-state stabilizer of sickle cell hemoglobin. ACS Med. Chem. Lett..

[13] Oksenberg D (2016). GBT440 increases haemoglobin oxygen affinity, reduces sickling and prolongs RBC half-life in a murine model of sickle cell disease. Br. J. Haematol..

[14] Sinha N, Tsai C-J, Nussinov R (2001). A proposed structural model for amyloid fibril elongation: domain swapping forms an interdigitating β-structure polymer. Protein Eng. Des. Sel..

[15] Fermi G, Perutz MF, Williamson D, Stein P, Shih DT-b (1992). Structure-function relationships in the low-affinity mutant haemoglobin aalborg (Gly74 (E18)β → Arg). J. Mol. Biol..

[16] Oksenberg D (2016). GBT440 increases haemoglobin oxygen affinity, reduces sickling and prolongs RBC half-life in a murine model of sickle cell disease. Br. J. Haematol..

[17] Demirci S, Uchida N, Tisdale JF (2018). Gene therapy for sickle cell disease: An update. Cytotherapy.

[18] Kato GJ (2018). Sickle cell disease. Nat. Rev. Dis. Prim..

[19] Xu D, Lin SL, Nussinov R (1997). Protein binding versus protein folding: The role of hydrophilic bridges in protein associations 1 1edited by B. Honig. J. Mol. Biol..

[20] Lounnas V, Wade RC (1997). Exceptionally stable salt bridges in cytochrome P450cam have functional roles. Biochemistry.

[21] Marqusee S, Sauer RT (1994). Contributions of a hydrogen bond/salt bridge network to the stability of secondary and tertiary structure in λ repressor. Protein Sci..

[22] Pucci F, Bourgeas R, Rooman M (2016). High-quality thermodynamic data on the stability changes of proteins upon single-site mutations. J. Phys. Chem. Ref. Data.

[23] Hendsch ZS, Tidor B (1994). Do salt bridges stabilize proteins? A continuum electrostatic analysis. Protein Sci..

[24] Nakamura H (1996). Roles of electrostatic interaction in proteins. Q. Rev. Biophys..

[25] Kumar S, Nussinov R (2002). Close-range electrostatic interactions in proteins. ChemBioChem.

[26] Chu X (2012). Importance of electrostatic interactions in the association of intrinsically disordered histone chaperone Chz1 and histone H2A.Z-H2B. PLoS Comput. Biol..

[27] Luo H (2004). A novel sickle hemoglobin: Hemoglobin S-south end. J. Pediatr. Hematol. Oncol..

[28] Perutz MF, Wilkinson AJ, Paoli M, Dodson GG (1998). The stereochemical mechanism of the cooperative effects in hemoglobin revisited. Annu. Rev. Biophys. Biomol. Struct..

[29] Suhail M, Usmani S, Ahmad M (2023). A quantum chemistry background of sickle cell anemia and gaps in antisickling drug development. Eur. J. Chem..

[30] Adachi K, Konitzer P, Paulraj CG, Surrey S (1994). Role of leu-beta 88 in the hydrophobic acceptor pocket for Val-beta 6 during hemoglobin S polymerization. J. Biol. Chem..

[31] Ferrone FA, Ivanova M, Jasuja R (2002). Heterogeneous nucleation and crowding in sickle hemoglobin: An analytic approach. Biophys. J..

[32] Dash B, Archana Y, Satapathy N, Naik S (2013). Search for antisickling agents from plants. Pharmacogn. Rev..

[33] Marengo-Rowe AJ (2006). Structure-function relations of human hemoglobins. Baylor Univ. Med. Cent. Proc..

[34] Rotter MA, Kwong S, Briehl RW, Ferrone FA (2005). Heterogeneous nucleation in sickle hemoglobin: Experimental validation of a structural mechanism. Biophys. J..

[35] Martin, D. W. Structure and function of a protein–haemoglobin. *Harper’s Rev. Biochem. 19th ed. Martin DW, Mayes PA Rodwell VN eds. Calif. Lange Med. Publ.* (1983).

[36] Tsai C-J, Lin SL, Wolfson HJ, Nussinov R (1997). Studies of protein-protein interfaces: A statistical analysis of the hydrophobic effect. Protein Sci..

[37] Dill KA (1990). Dominant forces in protein folding. Biochemistry.

[38] Makhatadze GI, Loladze VV, Ermolenko DN, Chen X, Thomas ST (2003). Contribution of surface salt bridges to protein stability: Guidelines for protein engineering. J. Mol. Biol..

[39] Ueda Y, Nagel RL, Bookchin RM (1979). An increased Bohr effect in sickle cell anemia. Blood.

[40] Chng KZ (2021). Assessment of transient changes in oxygen diffusion of single red blood cells using a microfluidic analytical platform. Commun. Biol..

[41] Mairbäurl H (1994). Red blood cell function in hypoxia at altitude and Exercise. Int. J. Sports Med..

[42] Mulquiney PJ, Bubb WA, Kuchel PW (1999). Model of 2,3-bisphosphoglycerate metabolism in the human erythrocyte based on detailed enzyme kinetic equations1: In vivo kinetic characterization of 2,3-bisphosphoglycerate synthase/phosphatase using 13C and 31P NMR. Biochem. J..

[43] Jensen M, Bunn HF, Halikas G, Kan YW, Nathan DG (1973). Effects of cyanate and 2,3-diphosphoglycerate on sickling relationship to oxygenation. J. Clin. Investig..

[44] Poillon WN, Kim BC, Labotka RJ, Hicks CU, Kark JA (1995). Antisickling effects of 2,3-diphosphoglycerate depletion. Blood.

[45] Frewin R (2014). Biochemical aspects of anaemia. Clinical Biochemistry: Metabolic and Clinical Aspects.

[46] Pelley JW (2012). Protein structure and function. Elsevier’s Integrated Review Biochemistry.

[47] Tashi T, Prchal JT (2016). Polycythemia. Lanzkowsky’s Manual of Pediatric Hematology and Oncology.

[48] Vekilov PG (2007). Sickle-cell haemoglobin polymerization: Is it the primary pathogenic event of sickle-cell anaemia?. Br. J. Haematol..

[49] Eaton WA (2020). Hemoglobin S polymerization and sickle cell disease: A retrospective on the occasion of the 70th anniversary of Pauling’s science paper. Am. J. Hematol..

[50] Jensen FB (2004). Red blood cell pH, the Bohr effect, and other oxygenation-linked phenomena in blood O_2_ and CO_2_ transport. Acta Physiol. Scand..

[51] Engel ER, Howard AL, Ankus EJ, Rico JF (2020). Advances in sickle cell disease management. Adv. Pediatr..

[52] Hoppe C, Neumayr L (2019). Sickle cell disease. Hematol. Oncol. Clin. N. Am..

[53] Abdulmalik O (2020). VZHE-039, a novel antisickling agent that prevents erythrocyte sickling under both hypoxic and anoxic conditions. Sci. Rep..

[54] Zaugg RH, Walder JA, Klotz IM (1977). Schiff base adducts of haemoglobin. Modifications that inhibit erythrocyte sickling. J. Biol. Chem..

[55] Oder E, Safo MK, Abdulmalik O, Kato GJ (2016). New developments in anti-sickling agents: Can drugs directly prevent the polymerization of sickle haemoglobin in vivo ?. Br. J. Haematol..

[56] Pagare PP (2018). Rational design of pyridyl derivatives of vanillin for the treatment of sickle cell disease. Bioorg. Med. Chem..

[57] Stern W, Mathews D, McKew J, Shen X, Kato GJ (2012). A phase 1, first-in-man, dose-response study of aes-103 (5-HMF), an anti-sickling, allosteric modifier of hemoglobin oxygen affinity in healthy Norman volunteers. Blood.

[58] Lehrer-Graiwer J (2015). GBT440, a potent anti-sickling hemoglobin modifier reduces hemolysis, improves anemia and nearly eliminates sickle cells in peripheral blood of patients with sickle cell disease. Blood.

[59] Ali MA (2020). Efficacy and safety of recently approved drugs for sickle cell disease: A review of clinical trials. Exp. Hematol..

[60] Cimpeanu E, Poplawska M, Jimenez BC, Dutta D, Lim SH (2021). Allogeneic hematopoietic stem cell transplant for sickle cell disease: The why, who, and what. Blood Rev..

[61] Ferrone FA (2018). Targeting HbS polymerization. Semin. Hematol..

[62] Leibovitch JN (2022). l-glutamine, crizanlizumab, voxelotor, and cell-based therapy for adult sickle cell disease: Hype or hope?. Blood Rev..

[63] Bellantoni AJ, Mangoli A, Deel MD (2023). Hematology of childhood and adolescence. Encyclopedia of Child and Adolescent Health.

[64] Estepp JH (2018). Voxelotor (GBT440), a first-in-class hemoglobin oxygen-affinity modulator, has promising and reassuring preclinical and clinical data. Am. J. Hematol..

[65] Hutchaleelaha A (2019). Pharmacokinetics and pharmacodynamics of voxelotor (GBT440) in healthy adults and patients with sickle cell disease. Br. J. Clin. Pharmacol..

[66] Eaton WA, Bunn HF (2017). Treating sickle cell disease by targeting HbS polymerization. Blood.

[67] Shaanan B (1983). Structure of human oxyhaemoglobin at 2·1resolution. J. Mol. Biol..

[68] Tame JRH, Vallone B (2000). The structures of deoxy human haemoglobin and the mutant Hb Tyrα42His at 120 K. Acta Crystallogr. Sect. D Biol. Crystallogr..

[69] Ghatge MS (2016). Crystal structure of carbonmonoxy sickle hemoglobin in R-state conformation. J. Struct. Biol..

[70] Harrington DJ, Adachi K, Royer WE (1997). The high resolution crystal structure of deoxyhemoglobin S. J. Mol. Biol..

[71] Fan J-S (2013). Solution structure and dynamics of human hemoglobin in the carbonmonoxy form. Biochemistry.

[72] Richard V, Dodson GG, Mauguen Y (1993). Human deoxyhaemoglobin-2,3-diphosphoglycerate complex low-salt structure at 2·5 Å resolution. J. Mol. Biol..

[73] Sinha N, Kumar S, Nussinov R (2001). interdomain interactions in hinge-bending transitions. Structure.

[74] Chikezie PC, Ekeanyanwu RC, Chile-Agada AB (2020). Polymerization of deoxygenated sickle hemoglobin in the presence of fractionated leaf extracts of *Anacardium occidentale*, *Psidium guajava*, and *Terminalia catappa*. Bull. Natl. Res. Cent..

[75] Meuzelaar H, Vreede J, Woutersen S (2016). Influence of Glu/Arg, Asp/Arg, and Glu/Lys salt bridges on α-helical stability and folding kinetics. Biophys. J..

[76] Donald JE, Kulp DW, DeGrado WF (2011). Salt bridges: Geometrically specific, designable interactions. Proteins Struct. Funct. Bioinform..

[77] Lim S, Choi D, Jeong T, Han D (2023). Carboxylate-derived conductive, sodium-ion storable surface of Prussian blue with a stable cathode-electrolyte interface. J. Alloys Compd..

[78] Gallops CE, Yu C, Ziebarth JD, Wang Y (2019). Effect of the protonation level and ionic strength on the structure of linear polyethyleneimine. ACS Omega.

[79] Gad SC (2023). Phosphoric acid. Reference Module in Biomedical Sciences.

[80] Spainhour CB (2014). Phosphoric Acid. Encyclopedia of Toxicology.

[81] Verma C (2022). Corrosion and corrosion inhibition in acidic electrolytes.

[82] Callaghan R, George AM, Kerr ID (2012). 8.8 Molecular aspects of the translocation process by ABC proteins. Comprehensive Biophysics.

[83] Grønborg M, Jensen ON (2003). Phosphoprotein and phosphoproteome analysis by mass spectrometry. J. Chromatogr. Libr..

[84] Cho J, King JS, Qian X, Harwood AJ, Shears SB (2008). Dephosphorylation of 2,3-bisphosphoglycerate by MIPP expands the regulatory capacity of the Rapoport-Luebering glycolytic shunt. Proc. Natl. Acad. Sci..

[85] The kinetics of the carbon dioxide-carbonic acid reaction. *Philos. Trans. R. Soc. Lond. Ser. A, Contain. Pap. Math. Phys. Character* **232**, 65–97 (1933).

[86] Brandt MJ, Johnson KM, Elphinston AJ, Ratnayaka DD (2017). Chemical storage, dosing and control. Twort’s Water Supply.

[87] Retallack, G. J. Impact of Past Global Warming on Biodiversity. in *Encyclopedia of Biodiversity* 224–230 (Elsevier, 2007). doi:10.1016/B978-0-12-384719-5.00232-X.

[88] McManus, J. W. Coral Reefs. in *Encyclopedia of Ocean Sciences* 660–670 (Elsevier, 2001). doi:10.1016/B978-012374473-9.00090-4.

[89] Sher, E. Environmental Aspects of Air Pollution. in *Handbook of Air Pollution From Internal Combustion Engines* 27–41 (Elsevier, 1998). doi:10.1016/B978-012639855-7/50041-7.

[90] Högman CF (1998). Preparation and preservation of red cells. Vox Sang..

[91] Kvassman J, Pettersson G (1989). Mechanism of 1,3-bisphosphoglycerate transfer from phosphoglycerate kinase to glyceraldehyde-3-phosphate dehydrogenase. Eur. J. Biochem..

[92] Haller, R. G. & DiMauro, S. Metabolic and Mitochondrial Myopathies. in *Muscle* 1031–1041 (Elsevier, 2012). doi:10.1016/B978-0-12-381510-1.00075-2.

[93] Płoszczyca K, Czuba M, Chalimoniuk M, Gajda R, Baranowski M (2021). Red blood cell 2,3-diphosphoglycerate decreases in response to a 30 km time trial under hypoxia in cyclists. Front. Physiol..

[94] Shung K, Lee M, Reid J, Finch C (1979). Effects of oxygen tension and pH on the ultrasonic absorption properties of sickle cells. Blood.

[95] Ueda Y, Bookchin RM (1984). Effects of carbon dioxide and pH variations in vitro on blood respiratory functions, red blood cell volume, transmembrane pH gradients, and sickling in sickle cell anemia. J. Lab. Clin. Med..

[96] Erik van der Linden & Foegeding, E. A. Gelation. in *Modern Biopolymer Science* 29–91 (Elsevier, 2009). doi:10.1016/B978-0-12-374195-0.00002-1.

[97] Di Liberto G (2016). Dense red blood cell and oxygen desaturation in sickle-cell disease. Am. J. Hematol..

[98] Poillon WN, Robinson MD, Kim BC (1985). Deoxygenated sickle haemoglobin. Modulation of its solubility by 2,3-diphosphoglycerate and other allosteric polyanions. J. Biol. Chem..

[99] Biovia, D. S. Discovery studio modeling environment. at (2017).

[100] Dennington, R., Keith, T. A. & Millam, J. M. GaussView Version 6. at (2019).

[101] Kuhlman B, Bradley P (2019). Advances in protein structure prediction and design. Nat. Rev. Mol. Cell Biol..

[102] Lahiri P, Verma H, Ravikumar A, Chatterjee J (2018). Protein stabilization by tuning the steric restraint at the reverse turn. Chem. Sci..

[103] Zandarashvili L, Esadze A, Iwahara J (2013). NMR studies on the dynamics of hydrogen bonds and ion pairs involving lysine side chains of proteins. Adv. Protein Chem. Struct. Biol..

[104] Blumenthal I (2001). Carbon monoxide poisoning. J. R. Soc. Med..

[105] Noguchi C, Schechter A (1981). The intracellular polymerization of sickle hemoglobin and its relevance to sickle cell disease. Blood.

[106] Rotter M, Yosmanovich D, Briehl RW, Kwong S, Ferrone FA (2011). Nucleation of sickle hemoglobin mixed with hemoglobin A: Experimental and theoretical studies of hybrid-forming mixtures. Biophys. J..

